# Peripheral Saturation and Perfusion Index on the First Day of Life Play a Role in Early Discharge of Healthy Term Newborns

**DOI:** 10.1155/2022/2887312

**Published:** 2022-05-29

**Authors:** Serafina Perrone, Maurizio Giordano, Giuseppe De Bernardo, Mara Corradi, Giulia Cecconi, Ilenia Fontanarosa, Elisa Laschi, Giuseppe Buonocore, Susanna Esposito

**Affiliations:** ^1^Department of Medicine and Surgery, University of Parma, Parma, Italy; ^2^Department of Clinical Medicine and Surgery, University of Naples Federico II, Naples, Italy; ^3^Division of Pediatrics Neonatology and NICU, Ospedale Buon Consiglio Fatebenefratelli, Naples, Italy; ^4^Department of Molecular and Developmental Medicine, University of Siena, Siena, Italy

## Abstract

**Introduction:**

Pulse oximetry screening is a safe, feasible test, effective in identifying congenital heart diseases in otherwise well-appearing newborns. Uncertainties still persist on the most effective algorithm to be used and the timing of screening. The aim of this study was to evaluate the role of the pulse oximetry screening associated with the peripheral perfusion index performed in the first 24 hours of life for the early detection of congenital heart diseases and noncongenital heart diseases in the newborns.

**Materials and Methods:**

A prospective observational cohort study was conducted. The enrollment criteria were as follows: term newborns with an APGAR score >8 at 5 minutes. The exclusion criteria were as follows: clinical signs of prenatal/perinatal asphyxia or known congenital malformations. Four parameters of pulse oximetry screening were utilized: saturation less than 90% (screening 1), saturation of less than 95% in one or both limbs (screening 2), difference of more than 3% between the limbs (screening 3), and preductal peripheral perfusion index or postductal peripheral perfusion index below 0.70 (screening 4). The likelihood ratio, sensibility, specificity, and positive and negative predictive values for identification of congenital heart diseases or noncongenital heart diseases (suspicion of perinatal infection and any respiratory diseases) were evaluated.

**Results:**

The best predictive results for minor congenital heart disease were obtained combining screening 3 and screening 4 (*χ*^2^ (1) = 15,279; *p* < 0.05; OR = 57,900 (9,465–354,180)). Screening 2, screening 3, and screening 4 were predictive for noncongenital heart diseases (*χ*^2^ (1) = 11,550; *p* < 0.05; OR = 65,744 (10,413–415,097)). Combined screenings 2–4 were predictive for both congenital heart disease and noncongenital heart disease (*χ*^2^ (1) = 22,155; *p* < 0.05; OR = 117,685 (12,972–1067,648)).

**Conclusions:**

Combining peripheral saturation with the peripheral perfusion index in the first 24 hours of life shows a predictive role in the detection of minor congenital heart diseases and neonatal clinical conditions whose care needs attention.

## 1. Introduction

Affecting nearly 1% of live births, congenital heart diseases (CHDs) represent a leading cause of congenital birth defect, with an adverse impact on infant mortality and morbidity [1, 2]. About a quarter of CHD can be classified as a critical congenital heart disease (CCHD) and defined as a life-threatening condition requiring catheter-based intervention or heart surgery during the neonatal period, especially in the first week [1, 3]. The introduction of a screening program for early detection of CCHD has substantially improved the health outcomes [4, 5]. In 2011, the US Health and Human Service Secretary's Advisory Committee on Heritable Disorders in Newborns and Children recommended CCHDs to be added to newborn screening panel [6]; in July 2018, this screening became mandatory in the USA [7]. Since then, pulse oximetry screening (POS) has improved the early detection of many cyanotic defects and mandatory screening has been associated with decreased mortality from CCHD [4, 5, 7, 8]. Pulse oximetry screening has proven to be a safe, feasible test, effective in identifying, in otherwise well-appearing newborns, CHD undetected by prenatal ultrasounds. The test has a high specificity (99.9%, CI 99.7–99.9) and a moderate sensitivity (76.5%, CI 67.7–83.5) in early detection of CHD, which make it suitable for universal screening [9]. The rationale for using this method is that most CCHDs are associated with some degree of hypoxemia, which, however, may not be clinically evident with cyanosis [10]. Some studies have also reported the detection by POS of other life-threatening conditions, such us sepsis and pneumonia, as an additional advantage [3, 11, 12]. While there is strong evidence about the efficiency of the screening, uncertainties persist on the most effective algorithm to be used and the timing of screening (before or after the first 24 hours) [13–15]. A recent meta-analysis compared sensitivity and specificity of the screening performed at different time periods showing a significant higher rate of false-positive results when POS was carried out within 24 hours of life (0.42% vs 0.006%, *p*=0.027) [9]. The peripheral perfusion index (PPI) is a noninvasive assessment that reflects the ratio of pulsatile to nonpulsatile blood flow in peripheral tissue; lower PPI values correspond to reduced peripheral perfusion as occurring in conditions such as specific CHD that reduce the stroke volume in arterial circulation [16]. Studies in the neonatal population have highlighted the potential for the PPI to be used as an assessment tool in various aspects of infant's health [17]. In particular, studies have associated the PPI in the newborn period with subclinical chorioamnionitis [18], as a possible screening tool for the presence of congenital heart malformations [16, 19], as a predictor of low superior vena cava flow [20], and as a sign of improved tissue oxygenation following blood transfusion in preterm infants. Therefore, the PPI might have a theoretical role in improving the accuracy of POS for CHDs, but its potential role in improving the screening efficiency and the CHDs' early detection has not been yet clarified. This study aimed to evaluate the predictive role of the preductal and postductal saturation associated with the PPI performed between 6 and 24 hours of life in the early detection of CHDs and/or noncongenital heart diseases (NCHDs) in the newborns.

## 2. Materials and Methods

### 2.1. Participants

The prospective observational cohort study was carried out in the II Level Neonatal Unit of University Hospital. The study protocol was approved by the Affiliated Hospital of Medical University. Written informed consent was obtained from all patients. The privacy of all participants was protected. The study was performed in accordance with the standards of the International Committee on Harmonization on Good Clinical Practice and the revised version of the Declaration of Helsinki principles. The enrollment criteria were as follows: gestational age ≥37 weeks, birth weight ≥2500 grams, APGAR score >8 at 5 minutes, and less than 24 hours of life. The exclusion criteria were as follows: screening performed prior to 6 hours of life, needs of resuscitation at birth,newborns with clinical signs of prenatal/perinatal asphyxia (pH < 7 in the umbilical cord artery), and newborns with known congenital malformations/chromosomal anomalies.

### 2.2. Procedures and Instruments

POS screening was carried out by the midwifes with a Masimo SET pulse oximeter between 6 and 24 hours of life together with the daily nursing manoeuvres. The Masimo SET pulse oximeter determines and reveals heart rate (beat for minute), peripheral oxygen saturation (percentage), and PPI (absolute number). Two successive measurements were performed, one at the palm of the right hand (preductal) and one at the foot (postductal), and the preductal and postductal values of oxygen saturation were recorded. In addition to these parameters, the preductal and postductal values of the PPI were recorded.

The screenings were judged as abnormal according to the following criteria:

- Any oxygen saturation measure is <90% (in the initial screen or in repeat screens) (screening 1)
- Oxygen saturation is <95% in the right hand and foot on three measures, each separated by one hour (screening 2)
- A >3% absolute difference exists in oxygen saturation between the right hand and foot on three measures, each separated by one hour (screening 3)
- Preductal or postductal PPI values less than 0.7 in three consecutive registrations repeated every 30 minutes (screening 4)

Newborns with abnormal results on at least 1 of these screenings were considered as test positive, and echocardiography was performed (with an SP2442 phased probe by Esaote). Every newborn, after screening, received a daily neonatal clinical examination by the physicians on duty until discharge to identify any sign of CHDs, such as cyanosis, heart murmurs, polypnea, and weak femoral pulses. If abnormalities were detected during the examination, echocardiography and biochemical analysis were performed.

CHDs were considered as follows: ventricular defects, patent ductus arteriosus, atrial defects, and pulmonary hypertension. NCHDs were considered as follows: suspicion of perinatal infection based on clinical signs, such as polypnea, transitory tachypnea, and/or C-reactive protein>2 mg/dl at 48 h of life, and any respiratory diseases.

### 2.3. Statistical Analysis

A statistician carried out statistical analysis with IBM SPSS Statistics for Windows, v.25 (Armonk, NY, IBM Corp.). Normal distribution of data was analysed by the Kolmogorov–Smirnov test. Binary logistic regression was executed to evaluate the factors that can be predictive for CHDs and NCHDs. Bayes' theorem was performed to reveal the probability that a newborn with a positive screening test was affected by CHDs and/or NCHDs. The likelihood ratio, sensibility, specificity, and positive predictive value and negative predictive values were established if the screening test had good screening tools. Differences with *p* < 0.05 were considered statistically significant.

## 3. Results

A total of 2151 newborns were approached for screening. One hundred fifty-eight newborns were excluded because screening was done before 6 hours of life. In the final analysis, 1993 newborns were included. Baseline characteristics of the newborns are described in Table 1. During the observation period, only 1 CCHD occurred, which was a case of aortic coartation. Of 1993 enrolled newborns, 14 newborns failed the screening test and were affected by CHD or NCHD (Table 2). CHD occurred in 57 newborns (2, 86%). Screening 3 and screening 4 were predictive for CHD both alone and together. The best result was obtained by combining screening 3 and screening 4 (*χ*^2^ (1) = 15,279; *p* < 0.05; OR = 57,900 (9,465–354,180)). Newborns that failed one of these screening tests had a probability of 17% to be affected by CHD (Table 3). NCHD occurred in 27 newborns (1, 35%). Screenings 2, 3 and 4 were predictive for NCHD (*χ*^2^ (1) = 11,550; *p* < 0.05; OR = 65,744 (10,413–415,097)). Newborns that failed one of these screening tests had a probability of 53% to be affected by NCHD (Table 4). Finally, combined screenings 2–4 were predictive for CHD or NCHD (*χ*^2^ (1) = 22,155; *p* < 0.05; OR = 117,685 (12,972–1067,648)). Newborns that failed one of these screening tests had a probability of 88% to be affected by CHD or NCHD (Table 5).

## 4. Discussion

A recent meta-analysis reported a higher false-positive rate when the POS screening was performed within 24 hours than 48 hours after birth (0.42% vs 0.06%) [9]. The authors concluded that it is more appropriate to screen after 24 hours of age because first there is the risk of overtreating children, subjecting them to further investigations with a waste of resources. One of the main concerns about the high false-positive rate was about the increased need for specialist assessment (consisting mainly in echocardiography). Nonetheless, the rate of 0.8% test positivity in newborns undergoing ultrasound for an abnormal POS appeared favourable if compared with infants undergoing echocardiography for asymptomatic murmur [21]. In our study, we found 0.1% false-positive rate for screening 4. Screening 4 was negative in 1936 cases, of which 1934 were very negative and 2 were false positives. Of these 2 false-positive newborns, 1 had NCHD. Based on these data, it is important to balance a low false-positive rate with the help of timely diagnosis. A first-day screening can lead to a false positive rate of 0.8% but allows for early detection of significant pathological conditions, while POS after 24 hours has been associated with a lower false-positive rate of 0.04% but also with a higher number of CCHDs picked up after acute collapse [15, 21, 22]. An early screening before CCHD becomes symptomatic and may decrease its adverse consequences, such as cerebral underperfusion and hypoxia, organ failure, and mortality. However, in the mentioned review, the probability of having CHD was not calculated if the test was positive; furthermore, the screening was carried out only based on preductal and postductal saturation and not considering the perfusion index. In our study, only screenings 3 and 4 were predictive of CHD with a low sensitivity and high specificity but with a posttest probability of 17%. Moreover, screenings 2, 3, and 4 were predictive for NCHD with a sensitivity and specificity of 33% and 99.6% and with a posttest probability of 53%. Finally, the combination of preductal saturation, postductal saturation, and PPI demonstrated a good predictive role for CHD and NCHD when carried out before 24 hours of life, with sensibility and specificity of 17, 95%, and 99.9% and with a posttest positive probability of 88%. Other studies have pointed out a false-positive rate of 30–70% consisting of NCHD, such as respiratory and infectious diseases, some of which are potentially life-threatening conditions and might have advantage of an early diagnosis through POS [12, 21–25]. In fact, there is also a trend in our country toward shorter postnatal stay after birth; this tendency makes it crucial to identify as many pathological situations as possible in the first hours of life in order to ensure adequate management of the newborn in case of CHD or NCHD. Our data support screening in the first 24 hours of life because it is not selective only for CHD but also for NCHD. Moreover, a newborn who tests positive for this screening has an 88% probability to be affected with CHD or NCHD. The utility demonstrated by the combination of preductal saturation, postductal saturation, and PPI on the first day of life in the diagnosis of infections, as well as of other potentially life-threatening conditions and minor CHD, is relevant as they could have important clinical consequences if not identified promptly [3, 12, 13, 23]. As such, the combination of preductal saturation, postductal saturation, and PPI might be considered as a new, feasible, and cost-effective algorithm for screening newborns in the first 24 hours of life. Furthermore, the effectiveness of the test can acquire even more value when very early hospital discharge policies are adopted. The test, in fact, allows the early identification of newborns in whom the foetal-neonatal transition has not yet fully occurred and in which an early identification of more- or less-severe conditions could prevent progressive clinical deterioration and improve the outcome. The PPI is a noninvasive, feasible, and cost-effective method for real-time evaluation of peripheral perfusion. Its value is indicated in the same instrument that detects the peripheral oxygen saturation. Although determined at the same time as SpO_2_, the PPI is calculated regardless of the patient's SpO_2_ level. The PPI is derived from the photoelectric plethysmographic signal of the transcutaneous oximetry and provides information on blood vessel function. The innovation of this article is to bring together the information derived from the pulse oximeter and perfusion index parameters. Adding PPI to routine screening for CHDs and NCHDs in the first 24 hours after birth in our research study improved screening sensitivity, and this is the first study that we know to report these results. The PPI has been proposed as a predictor of high-severity illnesses in neonates and has been reported to show the early postnatal changes in peripheral circulation of newborns; being affected by a reduction in stroke volume, low PPI values are expected in CCHDs [14, 16, 26–28]. However, PPI values potentially indicative of CCHD vary in the literature [16, 29, 30]. Granelli et al. reported that a PPI value of 0.7 (corresponding to the 5^th^ centile) or even lower is not itself significant when not correlated with other clinical signs. The PPI can in fact be influenced, on the first day of life, from conditions such as low body temperature and eventual physiological acrocyanosis [16]. According to a recent study, PPI measurements combined with pulse oximetry and clinical data are useful for the early identification of obstructive lesions of the left heart; however, the literature data are not yet conclusive and, indeed, other authors state that the perfusion index cannot be currently recommended as an additional newborn screening for CCHD [28, 30, 31]. In our study, the PPI increased the predictive role of POS in detecting pathological conditions and the risk of false positives appeared to be reasonable if compared with the risk of missing diagnosis of life-threatening diseases. The early use of both the preductal and postductal PPI in association with preductal and postductal oxygen saturation could offer an ulterior advantage, improving both the detection of CHDs and NCHDs. More specifically, this association is likely to be useful for identification of CHDs and respiratory and infectious illnesses that would otherwise go undetected or detected in an advanced stage when already symptomatic. The results of this study enhance evidence that indicates the potential benefits of the introduction of predischarge screening (comprehensive of preductal and postductal oxygen saturation and the PPI) as a routine procedure, especially in the case of discharge within 24 hours of life. This might have significant implications in clinical practice, as many perinatal services aim to support mothers and infants to go home from hospital during the first day of life after an uncomplicated delivery.

## 5. Conclusion

Preductal and postductal oxygen saturation in association with the PPI in the first 24 hours of life improve the predictive role of the POS in the detection of CHD and may assist clinicians in early identification of newborns that could be affected by other relevant clinical conditions, such as infection or respiratory disease. Further validation with more data is required.

## Figures and Tables

**Table 1:** Clinical characteristics of the enrolled population.

	Minimum	Maximum	Mean	Standard deviation
Gestational age	37	42 + 2	39.6	1.2
Hours of life	6.00	24.00	14.970	5.564
Preductal SaO_2_	92	100.00	98.521	1.415
Postductal SaO_2_	91	100.00	99.172	1.101
Delta prepost	0.00	7	1.004	1.020
Preductal PPI	0.30	8.30	1.697	0.800
Postductal PPI	0.20	13.10	1.583	0.754

**Table 2:** Newborns tested positive at the pulse oximeter screening.

N	Gestational age (weeks)	Age at screen (hours)	Criteria for failed screen in first result				PCR	Final diagnosis
			<90%	>3%	<95% one or both	PI < 0.7	>(92 mg/dl) at 48 hours	
1	38 + 3 days	23				x		DIV
2	38 + 4 days	22.5		x				Transient neonatal tachypnea
3	39 + 5 days	12		x			x	Risk of neonatal infection
4	39 + 5 days	9		x			x	PFO, risk of neonatal infection
5	39 + 6days	20				x		DIA
6	40 days	18			x		x	Risk of neonatal infection
7	40 + 2 days	15				x	x	Risk of neonatal infection
8	40 + 5 days	7			x		x	Risk of neonatal infection
9	40 + 5 days	18		x	x		x	Risk of neonatal infection
10	40 + 5 days	18.5		x				PDA
11	41 days	22		x	x			Negative
12	41 + 1 days	23.5		x				PDA
13	41 + 3 days	20				x	x	DIV, PDA, risk of neonatal infection
14	41 + 5 days	22			x		x	Risk of neonatal infection

DIA: interatrial defect; PDA: patent ductus arteriosus; PFO: patent foramen ovale; DIV: interventricular defect.

**Table 3:** Four modalities of screening in relation to congenital heart disease.

Predictive factors	Sensibility (%)	Specificity (%)	PPV (%)	NPP (%)	LR^+^	Posttest positive probability (%)	LR^−^	Posttest negative probability (%)	*χ* ^2^ test	DF	*p*value	OR (95% CI)
Screening 1	0	100	—	97	—	—	1	99	—	—	—	—
Screening 2	1.45	99.7	16.7	97	—	—	1	99	4, 129	1	>0.05	6, 896 (0.793–60,004)
Screening 3	7	99.8	50	97	35	22	0.93	99	64, 247	1	<0.05	36, 453 (8,877–149,685)
Screening 4	5.26	99.9	60	97	52.6	30	0.95	99	58, 908	1	<0.05	53, 722 (8,796–328,107)
Screenings 3 + 4	12.28	99.5	43.8	97	24.6	17	0.88	99	15, 279	1	<0.05	57, 900 (9,465–354,180)

DF = degree freedom; OR = odds ratio; PPV = positive predictive value; NPP = negative predictive value.

**Table 4:** Four modalities of screening in relation to noncongenital heart disease.

Predictive factors	Sensibility (%)	Specificity (%)	PPV (%)	NPP (%)	LR+	Posttest positive probability (%)	LR⁻	Posttest negative probability (%)	*χ*2 test	DF	p value	OR (95% CI)
Screening 1	0	100	0	98.7	0	0	1	99	—	—	—	—
Screening 2	14.8	99.9	66.7	98.8	148	67	0.85	98	192, 094	1	*p* < 0.05	170, 783 (29,783–979,298)
Screening 3	14.8	99.8	50	98.8	74	50	0.85	98	142, 228	1	*p* < 0.05	85, 304 (20,098–362,059)
Screening 4	7.4	99.8	40	98.7	37	34	0.93	99	56, 017	1	*p* < 0.05	52, 347 (8,379–327,031)
Screenings 2 + 3 + 4	33	99.6	56	99	82.5	53	0.67	98	11, 550	1	*p* < 0.05	65, 744 (10,413–415,097)

DF = degree freedom; OR = odds ratio; PPV = positive predictive value; NPP = negative predictive value.

**Table 5:** Four modalities of screening in relation to CHD and NCHD.

Predictive factors	Sensibility (%)	Specificity (%)	PPV (%)	NPP (%)	LR^+^	Posttest positive probability (%)	LR^−^	Posttest negative probability (%)	*χ* ^2^ test	DF	*p* value	OR (95% CI)
Screening 1	0	100	0	96	—	—	1	96	—	—	—	—
Screening 2	6.4	99.95	83.3	96.3	128	84	0.94	96	100, 941	1	<0.05	131, 096 (15,123–1136,430)
Screening 3	8.9	99.95	87.5	96.4	178	87.9	0.91	96	149, 231	1	<0.05	188, 704 (22,909–1554,358)
Screening 4	5.13	99.95	80	96.3	102.6	81	0.95	96	77, 166	1	<0.05	103, 459 (11,423–937,067)
Screenings 2 + 3 + 4	17.95	99.9	87.5	96.8	179.5	88	0.82	95	22, 155	1	<0.05	117, 685 (12,972–1067,648)

DF = degree freedom; OR = odds ratio; PPV = positive predictive value; NPP = negative predictive value.

## Data Availability

The data used to support the findings of this study are available from the corresponding author upon request.

## Abbreviations

CHDs: Congenital heart diseases
CCHDs: Critical congenital heart diseases
POS: Pulse oximetry screening
PPI: Peripheral perfusion index
NCHDs: Noncongenital heart diseases.

## Additional Points

Pulse oximetry screening during the first 24 hours of life leads to a high number of false positives. The peripheral perfusion index improves pulse oximetry posttest positive probability in the first 24 hours of life.The new screening algorithm in the first 24 hours of life identifies early neonatal clinical conditions whose care needs attention.
